# Miscellanies

**Published:** 1820-12-01

**Authors:** 


### MISCELLANEOUS INTELLIGENCE.

Mr. SWAN, surgeon to the Lincoln Hospital, has in the press, an account of a new method of making dried anatomical preparations so as to present the same appearances as a fresh subject when first dissected. *Second Edition, enlarged.*

Dr. M'CARTHY's communication on Enteritis is transmitted to the Editor of the LONDON MEDICAL REPOSITORY, as our plan forbids us inserting original papers. It will afterwards be noticed in our *Supplemental Review*, when glancing at the original departments of cotemporary journals.

We are informed by our friend and correspondent, Dr. JAMES KENNEDY, of Dunning, in Perthshire, that the Cæsarian operation has been performed in that neighbourhood a few weeks ago. The child is saved, but the mother died twenty-two hours after the operation. It must be recollected, however, that in this, and probably in most other cases in this country, the operation was performed at too late a period. The woman had been exhausted by *seven* days labour.

Some cases of bronchocele have been lately treated by seton, in this country, the plan recommended by Dr. QUADRI of Naples, and with success.

Dr. QUADRI found, by repeated dissections of goitres, that the ramifications of the thyriod arteries, *in the diseased gland*, were always very small, and consequently that the danger from hæmorrhage was quite imaginary.

